# Green Synthesis of Molecularly Imprinted Polymers for Selective Extraction of Protocatechuic Acid from Mango Juice

**DOI:** 10.3390/foods13182955

**Published:** 2024-09-18

**Authors:** Liping Zhang, Xin Song, Yuxiao Dong, Xiyan Zhao

**Affiliations:** School of Basic Medicine and Forensic Medicine, Henan University of Science and Technology, Luoyang 471000, China; s2036558171@163.com (X.S.); yxd2020871071@163.com (Y.D.); xyz20040816@163.com (X.Z.)

**Keywords:** molecularly imprinted polymer, deep eutectic solvent, chitosan, green synthesis, protocatechuic acid

## Abstract

A novel and environmentally friendly molecularly imprinted polymer (PCA-MIP) was successfully synthesized in an aqueous solution for the selective extraction of protocatechuic acid (PCA). In this study, a deep eutectic solvent (DES, choline chloride/methacrylic acid, 1:2, mol/mol) and chitosan were employed as the eco-friendly functional monomers. These two components interacted with PCA through hydrogen bonding, integrating a multitude of recognition sites within the PCA-MIP. Thus, the resulting PCA-MIP exhibited outstanding adsorption performance, rapid adsorption rate, and better selectivity, with a maximum binding capacity of 30.56 mg/g and an equilibrium time of 30 min. The scanning electron microscope (SEM) and Brunauer–Emmett–Teller (BET) analyses revealed that the synthesized polymers possessed a uniform morphology and substantial surface areas, which were conducive to their adsorption properties. Moreover, the PCA-MIP integrated with HPLC demonstrated its efficacy as an adsorbent for the selective extraction of PCA from mango juice. The PCA-MIP presented itself as an exemplary adsorbent, offering a highly effective and eco-friendly method for the enrichment of PCA from complex matrices.

## 1. Introduction

Protocatechuic acid (PCA), a member of natural phenolic acids, is extensively found in a variety of vegetables and fruits. Extensive pharmacological research has demonstrated that PCA exhibits significant anti-inflammatory, antioxidant, tumor-inhibitory, and neuroprotective properties [1,2,3]. Nowadays, PCA is predominantly extracted from foods and natural products [4,5]. However, due to the intricate composition of food matrices and interference from other compounds, conventional enrichment techniques for PCA are cumbersome, solvent-consuming, and make it difficult to achieve high-purity products [6]. Consequently, it is essential to develop novel adsorbent materials for the selective enrichment and extraction of PCA from complex crude extracts prior to chromatographic analysis.

Molecular imprinting is an effective method for the fabrication of selective adsorbents [7,8,9]. The molecularly imprinted polymer (MIP) is prepared through a polymerization process that incorporates a template, functional monomer, and crosslinker agent. After removal of template molecules via an appropriate method, the resultant polymer is endowed with imprinting cavities that are complementary to the spatial structure of the template [10]. These “cryo-memory sites” could again selectively recognize templates from complex matrices through hydrogen bonding, hydrophobic effects, or electrostatic interactions [11,12]. The main advantage of MIPs lies in their exceptional selectivity and affinity for the target molecules, similar to the well-known “lock-and-key” model. Furthermore, MIPs are characterized by their minimal preparation cost and higher physicochemical stability.

Currently, a variety of MIPs have been reported for their efficacy in the extraction and detection of PCA from complex samples. Xie et al. (2015) synthesized magnetic MIPs, with acetonitrile utilized as the dispersion medium, acrylamide serving as the functional monomer, and ethylene glycol dimethacrylate acting as the crosslinker, which exhibited impressive recoveries and minimal detection limits for the selective extraction of PCA from plant extracts [13]. Denderz et al. (2014) introduced two MIPs for gallic acid (GA) and PCA, crafted with methacrylic acid as the monomer and methanol as the porogen agent. These MIPs had been successfully applied as sorbents, facilitating the precise quantification of targeted phenolic compounds within red wine matrices [14]. Li et al. (2015) first fabricated the magnetic hollow porous MIPs (HPMIPs) for PCA in the presence of 4-vinylpyridine and glycidilmethacrylate as a co-monomer [15]. However, the constituent components such as functional monomers (acrylic acid, acrylamide, vinyl pyridine, etc.), crosslinking agents (ethylene glycol dimethacrylate and methylene-bis-acrylamide), and porogen agents (methanol, acetonitrile, chloroform) all have some toxicity, limiting the application of MIPs in the food, therapeutic, and pharmaceutical industries. Consequently, the synthesis of MIPs based on green chemistry strategies has garnered significant interest [16,17]. The core principle entails striving to minimize or avoid the utilization of toxic, hazardous, and volatile organic solvents as well as monomers, and instead adopting some green, safe, and biodegradable materials and methods, thereby mitigating the detrimental impacts on the environment and human health [18].

Deep eutectic solvents (DESs) constitute a class of self-assembling eutectic fluids composed of hydrogen bond acceptors and hydrogen bond donors in a predefined ratio [19]. DES, as an emerging eco-friendly solvent, has been successfully applied in the preparation of MIPs [20,21]. Compared with conventional functional monomers, the abundant functional groups inherent in DESs could bind to the template through hydrogen bonding interactions, thus augmenting the affinity and selectivity of MIPs [22,23]. Furthermore, DESs have been demonstrated to serve as crosslinking agents, thereby conferring enhanced rigidity to the polymeric structure [24]. By leveraging the malleable properties of DESs, poorly soluble template molecules can be transformed into DESs, which can then be used as both a monomer and a template for the preparation of MIPs [25,26]. Moreover, DESs-based MIPs exhibit commendable compatibility with aqueous media and provide excellent recognition performance for templates in aqueous solution [27,28]. Therefore, the combination of DESs and MIPs has provided a new idea for the development of eco-friendly materials.

Moreover, chitosan (CS) is widely recognized as an environmentally friendly natural polysaccharide endowed with the virtues of nontoxic, biocompatible, and biodegradable advantages [29]. Recently, CS has demonstrated significant potential application value in MIP synthesis [30]. Typically, CS inherently contains a multitude of functional groups, such as glycosides, hydroxyl, and amino, rendering it an ideal functional monomer for the binding of templates in ion-imprinted polymers [31]. Additionally, chitosan can serve as a crosslinker or a supportive matrix, thereby reinforcing the rigid structure of MIPs and augmenting their specific surface area [32,33]. The chitosan-based MIPs have received considerable attention for the micro-solid phase extraction target from complex matrix [34,35,36]. However, it is noteworthy that several MIPs incorporate some toxic reagents during their synthesis. For instance, Laskar et al. (2021) synthesized chitosan-based magnetic molecularly imprinted polymers utilizing an acetonitrile/toluene (75/25, *v*/*v*) mixture as a porogenic solvent for separation and enrichment of tricyclazole from rice and water samples [37]. Despite organic solvents like acetonitrile and toluene being commonly utilized in the synthesis of MIPs, their potential hazards to health and the environment cannot be overlooked. This study presents a novel approach for PCA-MIP synthesis using environmentally friendly DESs and chitosan as functional monomers to improve adsorption performance while minimizing environmental impact.

In this study, we designed and synthesized an innovative PCA-MIP via green synthesis strategies in an aqueous solution, using DESs and CS as the co-functional monomers and glutaraldehyde as the crosslinking agent. The structural features of PCA-MIP were also meticulously examined. The preparation conditions, adsorption performance, selectivity, and reusability of PCA-MIP were further validated through a series of adsorption experiments. Moreover, the applicability of PCA-MIP in combination with HPLC for the targeted extraction and precise detection of PCA from the crude extracts of mango juice is also demonstrated. This integration showcases the practical utility and selectivity of PCA-MIP in real samples.

## 2. Materials and Methods

### 2.1. Materials and Reagents

Protocatechuic acid (PCA, ≥97.0%), gallic acid (GA, ≥98%), ethyl gallate (EG, ≥99%), naringin (NRG, ≥98%), chitosan (CS), and glutaraldehyde (Glu, 50%) were obtained from Macklin (Shanghai, China). Methacrylic acid (MAA, AR) was obtained from the Damao Chemical Reagent Factory (Tianjin, China). Choline chloride (ChCl, ≥98%) was obtained from Yuanye Biotechnology (Shanghai, China). Acetic acid and methanol were supplied by Yongda Chemical Reagent (Tianjin, China).

### 2.2. Preparation of PCA-MIP

**Figure 1:**
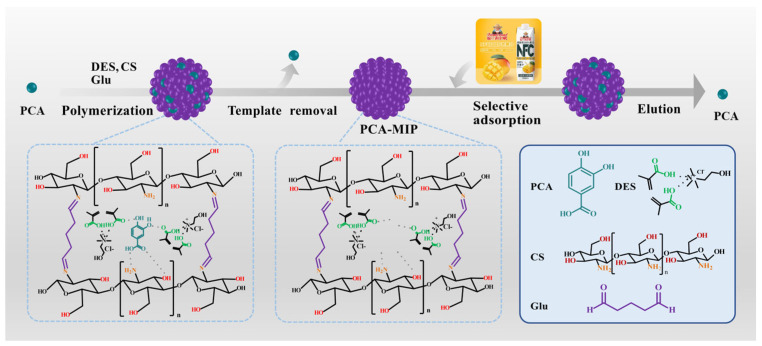
Schematic illustration of the procedure for the preparation of PCA-MIP.

Initially, the synthesis of DES was conducted by heating and mechanically stirring the ChCl and MAA (ChCl/MAA, 1/2, molar ratio) mixture for 2 h at 80 °C. This process culminated in the formation of a homogeneous and clarified liquid. Figure 1 illustrates the preparation process for PCA-MIP. The CS solution was obtained by dispersing 500 mg of CS into 50 mL of acetic acid solution (1%) and stirring to dissolve. Subsequently, 100 mg of PCA was dissolved in an appropriate amount of DES, and this solution was then gently immersed into 20 mL of prepared CS solution. The mixture was stirred for 1 h to ensure thorough mixing. An appropriate volume of Glu (0.5, 1.0, 2.0, and 3.0 mL), used as a crosslinking agent, was then added to the mixture and further stirred to ensure complete homogenization. Nitrogen gas was subsequently purged through the mixture to remove oxygen—a critical step to prevent unwanted side reactions during the polymerization process. The pre-polymerization mixture was then carefully transferred to a water bath at 65 °C and stirred continuously for 3 h to facilitate the polymerization reaction. The resulting polymeric matrix was evenly divided into centrifuge tubes and eluted with methanol and acetic acid mixture (9:1, *v*/*v*) until no trace of PCA was detectable by UV-vis spectrophotometry (UV-1000, Techcomp, Shanghai, China). Ultimately, the PCA-MIP was eluted three times with methanol and then dried under a vacuum. As a comparative control, the preparation process for non-imprinted polymer (PCA-NIP) was the same, but without PCA.

### 2.3. Optimization of Synthesis Conditions

The synthesis conditions (content of DES and volume of Glu) for PCA-MIP and PCA-NIP were optimized using a series of binding experiments. Briefly, 10 mg of dried PCA-MIP and PCA-NIP, derived from different preparation conditions, were dispersed into 1.5 mL of PCA aqueous solution at a fixed concentration of 500 μg/mL, with pH adjusted to 5.0. The mixtures were then subjected to a speed-regulated oscillator, ensuring uniform mixing for 4 h at room temperature. After centrifugal separation, the supernatant’s PCA concentration was quantified using a UV-vis spectrophotometer, at an absorbance wavelength of 260 nm. All adsorption experiments were conducted in triplicate, and the amounts (Q, mg/g) of PCA adsorbed by PCA-MIP and PCA-NIP were calculated using Equation (1). The imprinting performance of polymers was rigorously assessed using the imprinting factor (IF), and the formula was presented as Equation (2).

(1) $$Q=\frac{(C_{0}-C_{e})\times V}{M}$$

(2) $$IF=\frac{Q_{PCA-MIP}}{Q_{PCA-NIP}}$$

Here, *C*_0_ (mg/mL) signifies the initial concentration of PCA in the solution, while *C_e_* (mg/mL) is the concentration at equilibrium. The volume of the PCA solution is represented by *V* (mL) and *M* (g) is the mass of the polymers. *Q_PCA−MIP_* and *Q_PCA−NIP_* (mg/g) are the bonding amounts of polymers PCA-MIP and PCA-NIP toward PCA, respectively.

### 2.4. Characterization of PCA-MIP

The morphological characteristics of PCA-MIP and PCA-NIP were meticulously examined using an S-4800 SEM (Hitachi, Tokyo, Japan). FT-IR spectroscopy was employed to record the infrared spectra of PCA-MIP and PCA-NIP using a NICOLET380 Fourier transform infrared spectrophotometer (Thermo Fisher Scientific, Inc., Waltham, MA, USA). Furthermore, nitrogen adsorption–desorption of polymers was quantitatively assessed using an Autosorb-IQ Brunauer–Emmett–Teller analyzer (Quantachrome Instruments, Florida, USA). Thermal stability was measured using a Q50 Thermogravimetric analyzer (V20.8 Build 34, TA Instruments, Wilmington, DE, USA).

### 2.5. PCA-MIP Binding Study

The effect of incubation solution pH on the adsorption properties of PCA-MIP and PCA-NIP was initially investigated. A series of PCA aqueous solutions with an initial concentration of 500 μg/mL were prepared using water at pH values of 3.0, 4.0, 5.0, and 6.0. Subsequently, several portions of 10 mg of dried PCA-MIP and PCA-NIP were immersed in 1.5 mL of the PCA aqueous solutions at varying pH levels and mixed for 4 h. After centrifugation, the concentrations of PCA were measured using a UV-vis at 260 nm. The adsorption capacity (*Q*, mg/g) of PCA-MIP and PCA-NIP at different pH values was defined using Equation (1).

For equilibrium adsorption experiments, precise quantities of 10 mg each of the PCA-MIP and PCA-NIP were each suspended in 1.5 mL of PCA aqueous solutions with initial concentrations varying from 100 μg/mL to 1000 μg/mL, with the pH of the incubation medium standardized at 5.0. After shaking for 4 h, the mixtures were centrifuged to separate, and the equilibrium concentration of PCA was ascertained by a UV-vis spectrophotometer. The equilibrium adsorption capacity was then determined using Equation (1).

For kinetic adsorption experiments, 10 mg of PCA-MIP and PCA-NIP was introduced into 1.5 mL of PCA aqueous solutions with an initial concentration of 500 μg/mL, with the pH of the adsorption medium again set to 5.0. The mixtures were shaken at ambient temperature and the PCA concentrations were measured at certain intervals (10, 30, 60, 90, 120, 150, 180, and 240 min) using a UV-vis spectrophotometer. The dynamic adsorption capacity at time t, denoted as *Q_t_* (mg/g), was determined using Equation (3).

(3) $$Q_{t}=\frac{(C_{0}-C_{t})\times V}{M}$$

In this equation, *C*_t_ (μg/mL) are the concentrations of PCA at the respective time intervals.

Furthermore, selectivity adsorption experiments were performed by determining the adsorption capacity of polymers of PCA and its structural analogs, namely GA, NRG, and EG. Briefly, 10 mg of PCA-MIP and PCA-NIP were incubated in 1.5 mL of GA, NRG, and EG aqueous solutions (500 μg/mL). Following shaking for 4 h, the contents of the competitive molecules absorbed by polymers were quantified individually by a UV-Vis spectrophotometer at wavelengths of 272 nm, 280 nm, and 275 nm.

### 2.6. Study of PCA-MIP Reusability

The reusability of PCA-MIP was evaluated by determining the adsorption capacity with several rebinding–regeneration cycles. Initially, 10 mg of PCA-MIP was introduced into 1.5 mL of PCA aqueous solution (500 μg/mL, pH 5.0) and mixed for 4 h. Then, the PCA-MIP was collected by centrifugation, and the concentration of PCA in the supernatant was measured by UV-vis spectrophotometry. The sedimented PCA-MIP was then regenerated through three cycles of elution with a methanol/acetic acid mixture (9:1, *v*/*v*) to remove PCA. The following adsorption–desorption PCA-MIP experiments were repeated five times.

### 2.7. Extraction of PCA from Mango Juice

NFC mango juice (Anyang Jingshantang Beverage Co., Ltd., Anyang, China) was purchased from a local market in Luoyang. A 40 mL volume of the mango juice was first centrifuged for 20 min at 8000 rpm. Then, 25 mL of the supernatant was carefully extracted twice using an equivalent volume of ethyl acetate via liquid–liquid extraction. Subsequently, the ethyl acetate extract was evaporated to yield a dry residue. The mango juice crude extract was obtained by dissolving the dry residue in 20 mL of aqueous solution with a pH of 5.0. For the adsorption process, 1.5 mL of the mango juice crude extract was incubated with 30 mg of PCA-MIP for 4 h. The supernatant was harvested after separation, and adsorbents were then washed once with water and three times with a mixture of ethanol and acetic acid at a volume ratio of 9:1. The resultant crude extract, collected supernatant, eluate, and PCA standard were all filtered through 0.22 μm nylon membranes for HPLC analysis. HPLC detection was performed on a LaChrom Elite system (Hitachi, Tokyo, Japan) consisting of an LC 2130 pump and an LC 2030 detector that connected a Kromasii-C18 column (4.6 mm × 250 mm). The mobile phase was methanol/0.1% phosphoric acid (5/95, *v*/*v*) with a flow rate of 1.0 mL/min. The injected sample volume was 20 μL and the detection wavelength was 280 nm.

## 3. Results and Discussion

### 3.1. Preparation of PCA-MIP

Figure 1 illustrates the preparation route for PCA-MIP. Prior to polymerization, the green DES, rich in functional groups, engaged in hydrogen bonding interactions with PCA. In addition, CS, endowed with a number of amino and hydroxyl groups, formed a host–guest complex with PCA through electrostatic attraction and multi-site hydrogen bonding. In the presence of Glu, the free amino groups in CS were effectively crosslinked by the reactive aldehyde groups of Glu, thereby enhancing the mechanical properties of the co-polymers [38]. After the elution of PCA, PCA-MIP with selective recognition sites for PCA was fabricated. In order to obtain high adsorption capacity for efficient recognition, some important synthetic conditions need to be further optimized during the preparation process.

**Figure 2:**
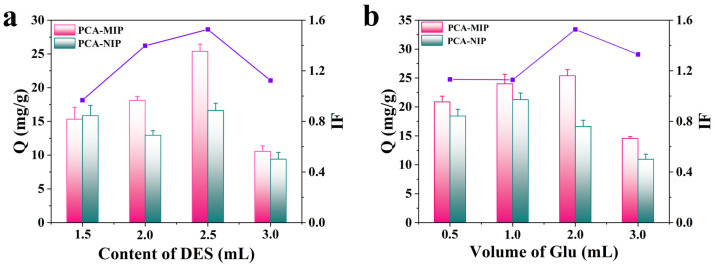
Effect of (**a**) content of DES and (**b**) volume of Glu on absorption capacity (Q) and imprinting factor (IF) of PCA-MIP and PCA-NIP. The pink bar represents PCA-MIP, the blue bar represents PCA-NIP, and the purple line is the imprinting factor.

In this system, the quaternary ammonium salt of CH_3_Cl mainly provided electrostatic interactions with PCA, and the HBD of MAA mainly produced hydrogen bonding interactions with PCA [20,39]. Therefore, the binding of CH_3_Cl with MAA can enhance its interaction with the PCA template through multiple interaction modes, resulting in the formation of more imprinting sites. As a consequence, the amount of DES directly affected the number of recognition sites within the polymer; thus, the content of DES was first investigated. As depicted in Figure 2a, the adsorption capacity of PCA-MIP showed an upward trend with the increment in DES content. When the DES content reached 2.5 mL, PCA-MIP achieved the highest adsorption capacity (Q = 25.39 mg/g) and the best imprinting performance (IF = 1.53)**.** When the content of DES was less than 2.5 mL, there were few imprinting cavities in MIP to bind more PCA. However, as the content of DES increased over 2.5 mL, both the adsorption amount and IF experienced a decline. The reason may be that steric hindrance from larger-sized polymers reduced adsorption capacity. In addition, the excessive addition of DES decreased the cross-section of the polymer network, which led to loose imprinting and lower binding capacity [40,41]. As a result, a DES volume of 2.5 mL was selected for the synthesis of PCA-MIP.

The role of the crosslinker was to maintain the rigid structure of the polymer matrix; therefore, the volume of Glu employed was critical in determining the degree of crosslinking within the PCA-MIP. From Figure 2b, it was found that the binding capacity and IF of PCA-MIP showed the same trend as the volume of Glu increased. At a Glu volume of 2.0 mL, the PCA-MIP demonstrated its optimal adsorption performance. This enhancement is postulated to arise from the increased Glu content, which facilitated the polymer to maintain a larger rigid structure, thereby better retaining the imprinted cavities and enhancing the recognition capability [42]. Nonetheless, when the volume of Glu exceeds 2.0 mL, both Q and IF dropped dramatically. The reason is that an excessive degree of crosslinking can lead to the embedding of imprinted sites, impeding the complete elution of the template and reducing specific adsorption of the polymer. Therefore, 2.0 mL of Glu was identified as the optimal crosslinker concentration.

### 3.2. Morphological and Structural Characterization of PCA-MIP

**Figure 3:**
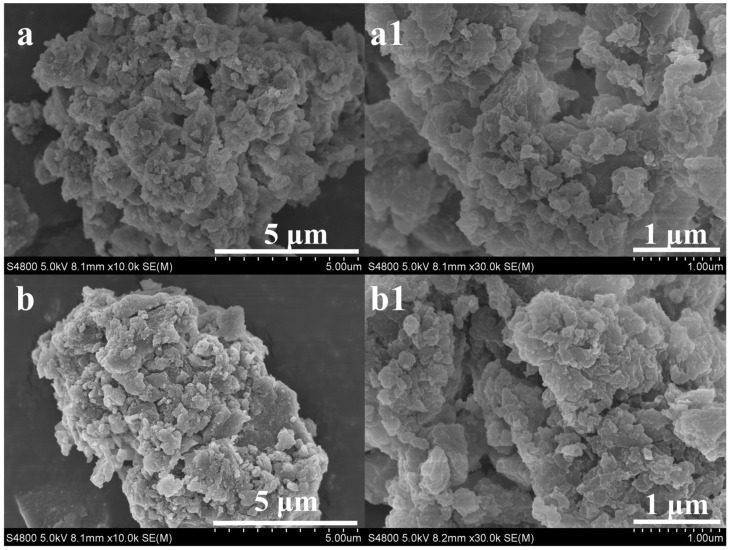
The SEM images of PCA-MIP (**a**,**a1**) and PCA-NIP (**b**,**b1**) with magnifications of 10,000× and 30,000×.

The morphological features of PCA-MIP and PCA-NIP were characterized by SEM. As illustrated in Figure 3a, the PCA-MIP exhibited a distinctive cauliflower-like surface structure, with particle sizes of 10–12 μm. In contrast, the PCA-NIP particles were clustered together to form irregular microparticles of approximately 7–10 μm (Figure 3b). Similarly, the SEM images at an increased magnification of 30,000× revealed that PCA-MIP possessed a relatively smooth and porous structure (Figure 3a1), whereas the PCA-NIP showed an irregular shape with some degree of adhesion (Figure 3b1). These differences were likely attributed to the incorporation of the template, and PCA-MIP retained numerous imprinted cavities after template elution. Conversely, PCA-NIP without the template resulted in a more amorphous aggregation.

**Figure 4:**
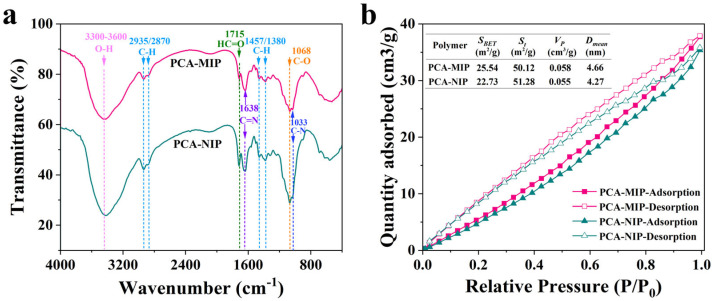
(**a**) FTIR spectra and (**b**) nitrogen adsorption–desorption isotherms of PCA-MIP and PCA-NIP. Inset in picture b are the pore parameters of PCA-MIP and PCA-NIP.

The FTIR spectra of PCA-MIP and PCA-NIP are illustrated in Figure 4a. It can be seen that both PCA-MIP and PCA-NIP present characteristic absorption peaks at 3300–3600 cm^−1^, corresponding to the O-H stretching vibration of DES and CS. The broader band in PCA-MIP proved hydrogen bonding formation between PCA and DES. The peaks at approximately 2935 cm^−1^, 2870 cm^−1,^ and 1457 cm^−1^, 1380 cm^−1^ were assigned to the C-H stretching vibration and C-H bending vibration of methyl and methylene groups in DES or Glu. The 1033 cm^−1^ band belonged to the stretching vibration of C-N in ChCl. These observations suggested that DES actively participated in the polymerization. Additionally, the strong adsorption peak near 1638 cm^−1^ was in agreement with the C=N stretching vibrations, indicative of Schiff base formation between CS and Glu [43]. The peaks at 1715 cm^−1^ were characteristic of the aldehyde group, probably from unreacted Glu. In addition, the strong peak at 1067 cm^−1^ was attributed to the stretching vibration of -C-O- within the furan ring of CS.

Nitrogen adsorption–desorption was carried out to assess the pore size distributions of PCA-MIP and PCA-NIP. As shown in Figure 4b, the BET isotherms exhibited a typical “type IV” pattern with H3 hysteretic loops within the relative pressure range of 0–1.0, indicating that PCA-MIP and PCA-NIP were mesoporous structures with narrow slit-like pores [44]. Further, the surface areas of the polymers were calculated using the Brunauer–Emmett–Teller (BET) equation. As shown in the inset in Figure 4b, PCA-MIP (25.54 m^2^/g) exhibited a greater surface area than that of PCA-NIP (22.73 m^2^/g), further substantiating the presence of imprinted sites within PCA-MIP. The pore size distribution (Figure S1) indicated that the pore volume of PCA-MIP was larger than that of PCA-NIP, with values of 0.058 cm^3^/g and 0.055 cm^3^/g, respectively. Moreover, the average pore sizes of the PCA-MIP and PCA-NIP were 4.66 nm and 4.27 nm, respectively, categorizing the synthesized polymers as mesoporous materials.

In order to investigate the thermal stability of PCA-MIP and PCA-NIP, thermogravimetric analysis (TGA) was performed. As shown in Figure S2, the two TGA curves were similar. Approximately 15.4% of mass loss was observed in the temperature range of 30 °C to 150 °C, probably due to evaporation of residual water within the adsorbent. There were two noticeable and drastic losses between 246 °C and 500 °C, which were mainly due to the decomposition of the polymer microsphere. As the temperature increased over 500 °C, the weight loss of both PCA-MIP and PCA-NIP remained relatively constant. It was worth noting that PCA-MIP had a weight loss of 63.5% at 500 °C, whereas PCA-NIP decomposed about 64.8% at the same temperature. These results indicate that PCA-MIP is more thermally stable than PCA-NIP.

### 3.3. PCA-MIP Binding Study

#### 3.3.1. Effect of pH on the Adsorption Capacity

**Figure 5:**
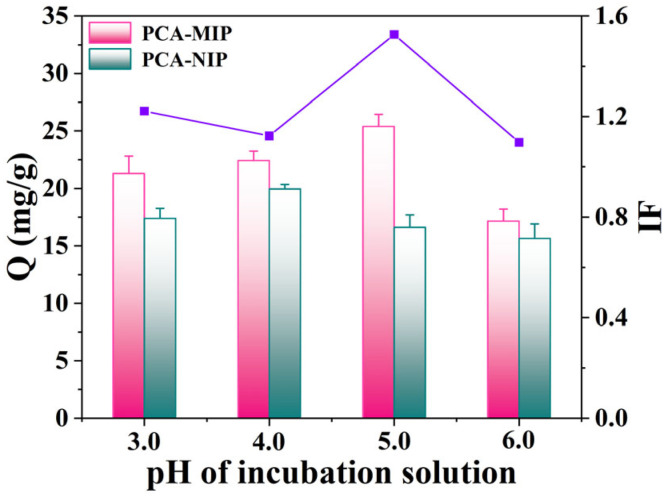
Effect of pH of incubation solution on absorption capacity (Q) and imprinting factor (IF) of PCA-MIP and PCA-NIP. The pink bar represents PCA-MIP, the blue bar represents PCA-NIP, and the purple line is the imprinting factor.

The pH value of the adsorption medium exerts a profound influence on both the existence state of PCA and the binding efficacy of PCA-MIP. To identify the optimal conditions for PCA adsorption, the pH of the adsorption medium was systematically investigated. Considering that the pKa value of PCA was 4.48, the incubation medium pH was selected from 3.0 to 6.0. As illustrated in Figure 5, the adsorption capacity of polymers gradually increased with pH increase from 3.0 to 5.0. Notably, the optimal imprinting performance of PCA-MIP with an imprinting factor of 1.40 was achieved at pH 5.0. This enhancement is primarily attributed to the neutrality of PCA at pH 5.0, allowing for the phenolic hydroxyl and carboxyl groups to effectively engage in multiple hydrogen bonding interactions with the complementary sites on PCA-MIP. Consequently, this alignment resulted in the high degree of adsorption of PCA-MIP for PCA [45]. However, upon surpassing pH 5.0, the adsorption capacity gradually decreased. This reduction may be attributed to the strong electrostatic repulsion or attraction between the ionized PCA and polymers, which weakened the adsorption process. Thus, the optimal pH of the incubation solution was 5.0 in the following experiments.

#### 3.3.2. Equilibrium Adsorption Isotherms

**Figure 6:**
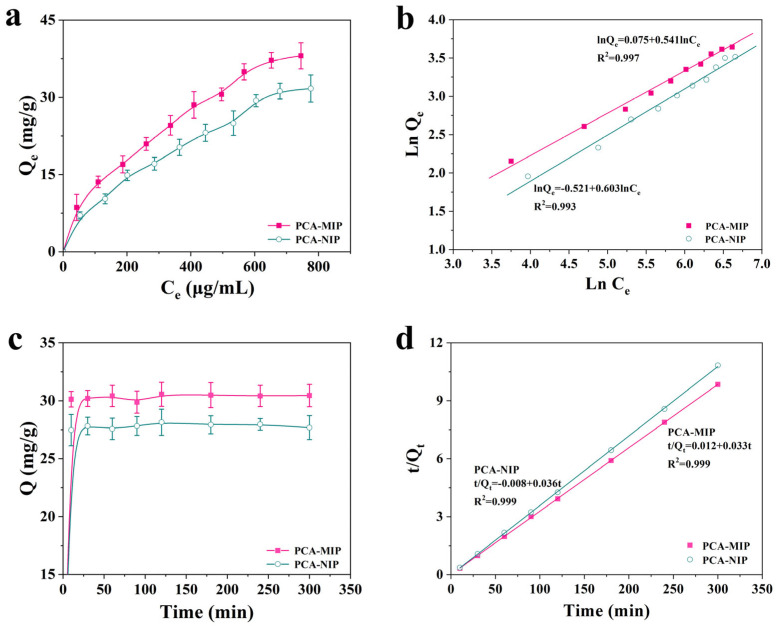
(**a**) Equilibrium adsorption curves, (**b**) linear fitting curves of Freundlich model, (**c**) adsorption kinetics curves, and (**d**) linear fitting curves of a pseudo-second-order kinetic model for PCA-MIP and PCA-NIP.

The equilibrium adsorption experiments were investigated at a pH of 5.0, with initial concentrations of PCA from 100 to 1000 μg/mL. As illustrated in Figure 6a, the adsorption capacities of PCA-MIP and PCA-NIP exhibited a gradual increase with escalating PCA concentrations, reaching saturation at approximately 800 μg/mL. The amount of binding PCA-MIP was always higher than that of PCA-NIP under the same initial concentrations, and their maximum amounts were 38.07 mg/g and 31.70 mg/g, respectively. The results underscored that the numerous binding sites within PCA-MIP could effectively recognize PCA during the adsorption process. To elucidate the adsorption mechanism, the equilibrium adsorption isotherms were analyzed using Langmuir models, Freundlich models, and Scatchard models (Equations (4)–(6)).

(4) $$\frac{C_{e}}{Q_{e}}=\frac{C_{e}}{Q_{max}}+\frac{1}{Q_{max}K_{L}}$$

(5) $$lnQ_{e}=lnK_{F}+\frac{1}{n}lnC_{e}$$

(6) $$\frac{Q_{e}}{C_{e}}=\frac{Q_{max}-Q}{K_{D}}$$

In these equations, *Q_e_* (mg/g) is the equilibrium adsorption capacity of PCA on polymers and *Q_max_* (mg/g) represents the maximum adsorption capacity. *C_e_* (mg/mL) is intended for the concentration of PCA at equilibrium. *K_L_* (L/mg) and *K_F_* (L/g) are the Langmuir and Freundlich constants, respectively, 1/n is the equilibrium adsorption index of the isotherm, and *K_D_* (mg/mL) is the dissociation constant.

The fitting curves and calculated parameters corresponding to the adsorption isotherms are detailed in Figure 6b and Figure S3 and Table 1. The Freundlich linear regression equations for PCA-MIP and PCA-NIP were expressed as lnQ_e_ = 0.541lnC_e_ + 0.075 (R^2^ = 0.997) and lnQ_e_ = 0.603 lnC_e_-0.521 (R^2^ = 0.993), respectively. The Langmuir equations for these polymers were determined to be C_e_/Q_e_ = 0.018C_e_ + 6.439 (R^2^ = 0.967) and C_e_/Q_e_ = 0.019C_e_ + 9.766 (R^2^ = 0.994). Obviously, the correlation coefficient (R^2^) values of the Freundlich model for PCA-MIP and PCA-NIP were greater than those of the Langmuir model, suggesting that the adsorption process of PCA-MIP was predominantly controlled by a multilayer adsorption mechanism [46]. Additionally, the Scatchard plot (Figure S4) for PCA-MIP was characterized by two distinct straight lines with varying slopes, suggesting the presence of both high-affinity specific binding sites and low-affinity non-specific binding sites for PCA. As detailed in Table 1, for the high-affinity imprinting sites for PCA-MIP, K_D_ and Q_max_ were calculated to be 0.074 mg/mL and 23.30 mg/g, respectively. However, the Scatchard plot for PCA-NIP displayed a single linear relationship, yielding K_D_ and Q_max_ values of 0.649 mg/mL and 58.83 mg/g, respectively, suggesting that PCA-NIP possessed only non-specific binding sites for PCA.

#### 3.3.3. Kinetic Adsorption

The kinetic adsorption profiles of PCA-MIP and PCA-NIP are displayed in Figure 6c. Initially, both PCA-MIP and PCA-NIP exhibited a swift rise in binding capacity, with an especially rapid increase observed within the first 10 min. Subsequently, equilibrium was attained at approximately 30 min. Notably, PCA-MIP demonstrated a superior adsorption capacity of 30.56 mg/g compared to PCA-NIP (27.68 mg/g). The faster adsorption rate and higher binding amount were attributed to the existence of specific imprinting sites in PCA-MIP that facilitated the capture and mass transfer of PCA. Furthermore, the data were fitted with the pseudo-first-order kinetic model (Equation (7)) and the pseudo-second-order kinetic model (Equation (8)).

(7) $$\ln\left(Q_{f}-Q_{t}\right)=lnQ_{f}-k_{1}t$$

(8) $$\frac{t}{Q_{t}}=\frac{1}{k_{2}Q_{s}^{2}}+\frac{t}{Q_{s}}$$

Here, *Q_f_* and *Q_s_* (mg/g) represent the adsorption amounts at equilibrium, and *k*_1_ and *k*_2_ denote the rate constants.

As depicted in Figure 6d and Figure S5 alongside Table 2, the higher R^2^ values demonstrated that the kinetic adsorption data for PCA-MIP exhibited a superior fit to the pseudo-second-order kinetic model (R^2^ = 0.999), surpassing that of the pseudo-first-order model (R^2^ = 0.671). In addition, the calculated adsorption amount from the pseudo-second-order kinetic model (Q_s_ = 30.49 mg/g) was in excellent agreement with the experimentally determined value of 30.56 mg/g. The results indicated that the process of PCA-MIP adsorption to PCA was a chemisorption process potentially involving a rate-limiting step [22].

#### 3.3.4. Selectivity Adsorption

**Figure 7:**
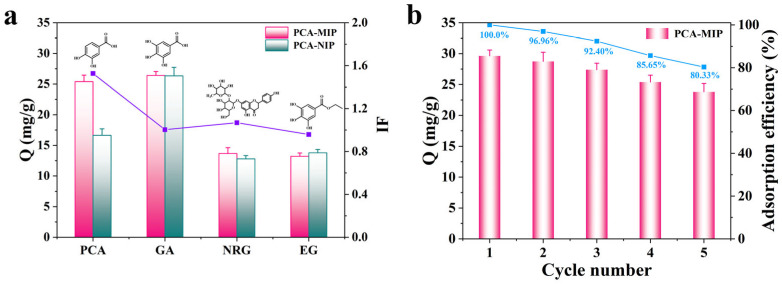
(**a**) Selective adsorption capacities of PCA-MIP and PCA-NIP for PCA and its analogs. (**b**) Reusability analysis of PCA-MIP via five sequential cycles of adsorption–desorption.

The selectivity of PCA-MIP was rigorously assessed by determining its adsorption capacity for PCA in comparison to its competitors, namely GA, NRG, and EG (structures shown in Figure 7a). From Figure 7a, it is notable that PCA-MIP presented a higher binding capacity for PCA (25.39 mg/g) compared to NRG and EG. This observation suggests that the specific imprinting sites within PCA-MIP conferred remarkable selectivity for PCA. Strangely, PCA-MIP also displayed higher adsorption capacity for GA, but with a slight imprinting factor. This is primarily attributed to the functional groups and structure of GA being similar to those of PCA and may enter the imprinted cavities of PCA-MIP during adsorption. Additionally, the presence of more phenolic hydroxyl groups in GA compared to PCA may enhance its nonspecific binding to the excess groups of CS. Therefore, the adsorption of GA onto PCA-MIP was predominantly a nonspecific adsorption process.

### 3.4. Reusability of PCA-MIP

To assess the reusability of PCA-MIP, a series of consecutive adsorption–desorption cycles were performed on the identical samples. As shown in Figure 7b, it was noted that a minor reduction in the adsorption capacity of PCA-MIP occurred, from 29.64 mg/g to 23.81 mg/g, while the adsorption efficiency remained consistently above 80.33% even after five cycles. The minor reduction in adsorption capacity might be ascribed to potential damage or blockage of the imprinting cavities during the elution process. By comparing with PCA-imprinted MIPs in Table 3, the preparation of PCA-MIP was facile, green, and time-efficient. In addition, PCA-MIP also demonstrated greater recognition ability than other MIPs [13,47,48,49]. Overall, the prepared PCA-MIP demonstrated satisfactory reusability and stability, making it a robust candidate for practical applications.

### 3.5. Extraction of PCA from Mango Juice

Recently, NFC mango juice, a beverage, has attracted more scientific attention due to its valuable health benefits as well as being rich in high levels of phenolic compounds [50]. It also acts as a major source of antioxidants such as PCA, phenolic acids, mango glycosides, quercetin, mango keto acid, isomangiferolic acid, and other biologically active substances [51]. However, due to its complicated matrix, the extraction and determination of the active components directly from mango juice are tedious and inefficient. Therefore, in order to reduce interference by other polar components, it is very necessary to employ a selective pretreatment step to separate the PCA from the mango juice.

PCA-MIP was expected to be an efficient adsorbent material and its ability to extract PCA from mango juice was evaluated. Initially, the HPLC method for the analysis of PCA was first established, encompassing linearity, correlation coefficients, limit of detection (LOD), and limit of quantification (LOQ). A linear calibration curve was obtained using the standard curve method. The HPLC peak area (A) for the corresponding PCA concentration (C) was found to be linear between 1 and 100 μg/mL. The linear equation was A = 23119C + 29874, with an R^2^ value of 0.998. The LOD and LOQ were determined to be 0.81 μg/mL and 2.68 μg/mL, respectively, based on the content relative to 3 and 10 times the baseline noise.

**Figure 8:**
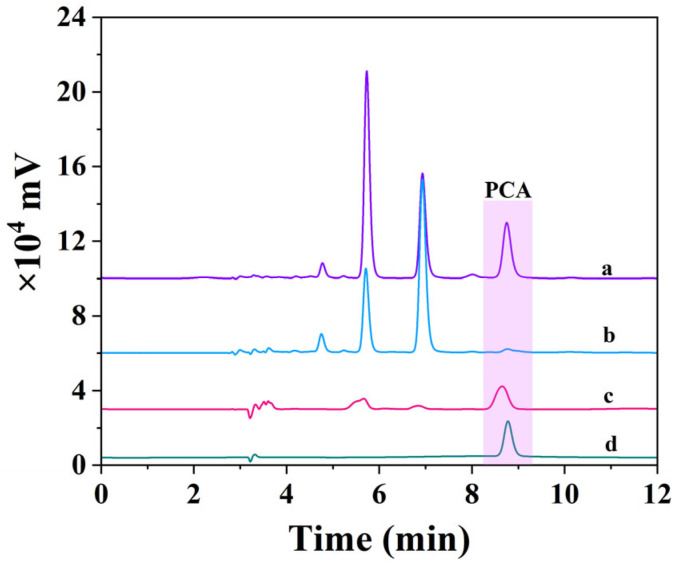
Chromatograms of (**a**) mango juice crude extract, (**b**) supernatant of mango juice crude extract from PCA-MIP, (**c**) elution of mango juice crude extract from DYM-MIP, and (**d**) PCA standard.

Furthermore, the chromatograms of mango juice crude extract, the supernatant and eluent of PCA-MIP, and a PCA standard sample (5 μg/mL) were recorded. As shown in Figure 8d, the retention time of the PCA standard was identified as 5.60 min while the interference peaks in mango juice crude extract also occurred at nearly the same retention time as PCA (Figure 8a), which directly affected the accuracy of PCA content determination. Following selective extraction by PCA-MIP, there was a significant reduction in PCA in the supernatant (Figure 8b), suggesting specific adsorption of PCA by the polymer. In Figure 8c, the PCA peaks were significantly enhanced and the interference peaks were nearly eliminated, resulting in extraction recoveries of PCA from mango juice ranging from 52.99% to 61.08%. These results clearly highlight the good practical applicability of fabricated PCA-MIP in the separation and enrichment of PCA from complex samples.

## 4. Conclusions

In summary, a novel type of PCA-MIP based on green synthesis strategies was prepared in an aqueous solution using DES and CS as the functional monomer and glutaraldehyde as the crosslinker. Compared with other MIPs, this work presented several distinct advantages: (1) the preparation strategy for PCA-MIP was facile, green, and time-efficient; (2) PCA-MIP exhibited excellent recognition ability and the adsorption capacity was greater than that of other MIPs; (3) PCA-MIP achieved a rapid mass transfer rate with an equilibrium time of 30 min. Thus, the resultant polymer presented outstanding adsorption performance, rapid adsorption rate, and better selectivity for PCA. Furthermore, PCA-MIP demonstrated great practical utility in the selective extraction of PCA from mango juice. Based on the research results and practicability potential, the following suggestions can be made: (1) Increasing the types of templates—more types of compounds can be selected, including proteins, peptides, and DNA, rather than only small molecule compounds. (2) Exploring more green functional monomers—more functional monomers containing specific functional groups should be explored to improve the adsorption capacity and imprinting performance of MIPs in aqueous environments. (3) Expanding the range of application—in addition to selective separation of PCA in the target juice, other complex products have been added that are more suitable for practical applications.

## Figures and Tables

**Table 1:** The fitting parameters for the Freundlich model and Scatchard analysis for PCA-MIP and PCA-NIP.

Polymer	*Q_exp_* (mg/g)	Freundlich Model			Low-Affinity Sites			High-Affinity Sites		
		*K_F_* (L/g)	*n*	R^2^	*K_D_* (mg/mL)	Q_max_ (mg/g)	R^2^	*K_D_* (mg/mL)	Q_max_ (mg/g)	R^2^
PCA-MIP	38.07	1.078	1.85	0.997	0.671	74.23	0.971	0.074	23.30	0.992
PCA-NIP	31.70	0.594	1.66	0.993	0.649	58.83	0.930	/	/	/

**Table 2:** The fitting parameters for pseudo-first-order and pseudo-second-order models for PCA-MIP and PCA-NIP.

Polymer	*Q_e_* (mg/g)	Pseudo-First-Order Model			Pseudo-Second-Order Model		
		*Q_f_* (mg/g)	K_1_ (mg/g/min)	R^2^	Q_s_ (mg/g)	K_2_ (g/mg/min)	R^2^
PCA-MIP	30.56	2.55	0.005	0.671	30.49	0.089	0.999
PCA-NIP	27.68	1.77	0.005	0.867	27.80	0.161	0.999

**Table 3:** Comparison of this work with other PCA-imprinted MIPs.

Polymer	Functional Monomer	Solvent	Synthesis Time (Hours)	Adsorption Capacity (mg/g)	Equilibrium Time (min)	Ref
MMIPs	4-vinylpyridine	Acetonitrile	24	7.5	40	[13]
Fe_3_O_4_@mSiO_2_@MIP	4-vinylpyridine	Acetonitrile	24	17.2	140	[47]
MMIPs	acrylamide	Acetonitrile	24	-	30	[48]
MIPs@Fe_3_O_4_-NH_2_	dopamine	Tris-HCl	3.5	46.48	50	[49]
PCA-MIP	DES and CS	Aqueous solution	3	30.56	30	This work

## Data Availability

The original contributions presented in the study are included in the article/Supplementary Materials, further inquiries can be directed to the corresponding author.

## Supplementary Materials

The following supporting information can be downloaded at: https://www.mdpi.com/article/10.3390/foods13182955/s1, Figure S1: Pore volumes of PCA-MIP and PCA-NIP; Figure S2: Linear fitting curves of the Langmuir model for PCA-MIP and PCA-NIP; Figure S3: Linear fitting curves of the Scatchard model for PCA-MIP and PCA-NIP; Figure S4: Linear fitting curves of the pseudo-first-order model for PCA-MIP and PCA-NIP. Figure S5: The liner fitting curves of Pseudo-first-order model for PCA-MIP and PCA-NIP.

## Abbreviations

PCA	Protocatechuic acid
DES	Deep eutectic solvents
CS	Chitosan
Glu	Glutaraldehyde
MAA	Methacrylic acid
ChCl	Choline chloride
MIP	Molecularly imprinted polymer
NIP	Non-molecularly imprinted polymer
SEM	Scanning electron microscope
BET	Brunauer–Emmett–Teller
FT-IR	Fourier transform infrared spectroscopy
HPLC	High-performance liquid chromatography
