# Supplementary material for: Green Synthesis of Molecularly Imprinted Polymers for Selective Extraction of Protocatechuic Acid from Mango Juice
Source: Foods. 2024 Sep 18;13(18):2955. doi: 10.3390/foods13182955 (PMC11431359; doi:10.3390/foods13182955)
Supplement: Supplementary file 1 [file foods-13-02955-s001.zip › foods-3185522-supplementary.pdf]

Supplementary Material

for

Green synthesis of molecularly imprinted polymer for the selective extraction of protocatechuic acid from mango juice

Liping Zhang* Xin Song Yuxiao Dong Xiyan Zhao

School of Basic Medicine and Forensic Medicine, Henan University of Science and Technology, Luoyang 471000, P.R. China

Correspondence: Liping Zhang, Henan University of Science and Technology, Luoyang, 471000, P.R. China

E-mail: lipingzhang1826@163.com

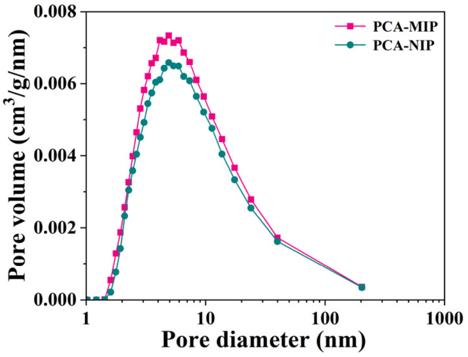

Figure S1. Pore volume of PCA-MIP and PCA-NIP.

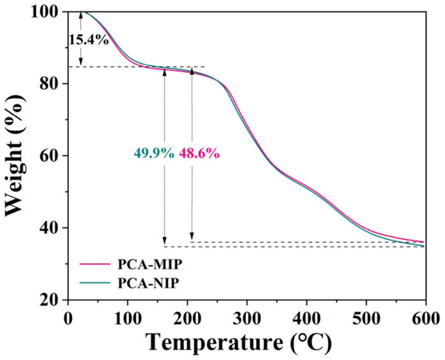

Figure S2. The TGA curves of PCA-MIP and PCA-NIP.

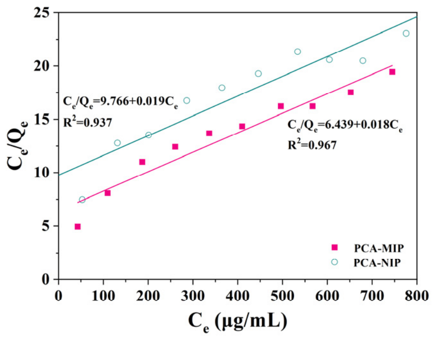

Figure S3. The liner fitting curves of Langmuir model for PCA-MIP and PCA-NIP.

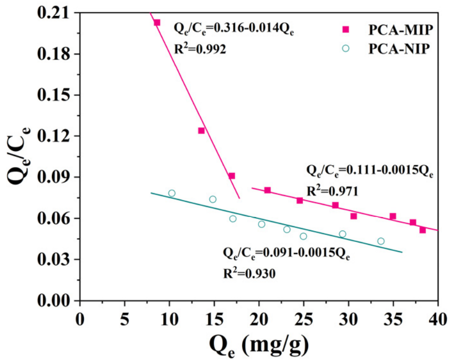

Figure S4. The liner fitting curves of Scatchard model for PCA-MIP and PCA-NIP.

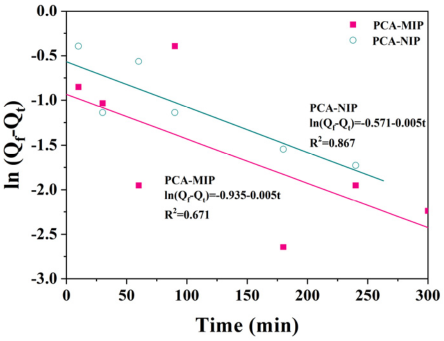

Figure S5. The liner fitting curves of Pseudo-first-order model for PCA-MIP and PCA-NIP.
